# *Leishmania major* Infection in Synanthropic Rodents: Evidence for the Urbanization of Zoonotic Cutaneous Leishmaniasis (ZCL) in Southern Iran

**DOI:** 10.1155/2024/4896873

**Published:** 2024-03-07

**Authors:** Saeed Shahabi, Kourosh Azizi, Qasem Asgari, Bahador Sarkari

**Affiliations:** ^1^Department of Biology and Control of Disease Vectors, School of Health, Shiraz University of Medical Sciences, Shiraz, Iran; ^2^Research Center for Health Sciences, School of Health, Shiraz University of Medical Sciences, Shiraz, Iran; ^3^Department of Parasitology and Mycology, School of Medicine, Shiraz University of Medical Sciences, Shiraz, Iran; ^4^Basic Sciences in Infectious Diseases Research Center, Shiraz University of Medical Sciences, Shiraz, Iran

## Abstract

Cutaneous leishmaniasis is of particular importance in southern Iran. This study aimed to investigate the infection of rodents with *Leishmania major* in an urban area of Fars Province, located in southern Iran. Rodents were trapped and samples from the liver, spleen, and skin were collected. Impression smears were prepared from these tissues and any skin lesions and were examined microscopically. In addition, a portion of the samples were preserved for subsequent DNA extraction. A total of 41 rodents belonging to three species were caught from 10 trapping stations in gardens or houses within the area. The caught rodent species were *Rattus rattus* (*n* = 25, 60.97%), *Mus musculus* (*n* = 15, 36.58%), and *Meriones persicus* (*n* = 1, 2.5%). *Leishmania* amastigotes were seen in the spleen tissue smear of 6 (2.43%) of the rodents, including 4 of *R. rattus* and 2 of *M. musculus*. Skin lesions were observed on the muzzles of two *R. rattus* and one *M. musculus*. Samples taken from these lesions tested positive for *Leishmania* infection. *Leishmania* DNA was detected in 18 (43.9%) rodents, including 11 *R. rattus,* 6 *M. musculus*, and one *M. persicus*, based on DNA sequencing of the ITS2 gene and PCR of the kDNA. Phylogenetic reconstruction revealed that the parasite infecting the rodents was *L. major*. The detection of *Leishmania* infection in these rodents in urban areas raises concerns about the urbanization of cutaneous leishmaniasis caused by *L. major*. This urbanization poses unique challenges for control and prevention efforts.

## 1. Introduction

Leishmaniasis, as one of the most neglected tropical diseases [1, 2], is a group of diseases including cutaneous, visceral, and mucocutaneous leishmaniasis caused by infection with protozoan parasites from over 20 *Leishmania* species. The disease is transmitted through the bite of infected sandflies. Cutaneous leishmaniasis (CL) is the most common form of the disease which causes skin lesions, especially in hands, feet, and faces [3–5]. CL is one of the most important parasitic diseases in Asian and Middle Eastern countries [6–9].

CL is a significant public health concern in Iran, with a long history of reported cases and a high prevalence rate [10–14]. The disease poses a considerable burden on the healthcare system, economy, and overall quality of life for affected people [15, 16].

CL holds particular significance in Fars Province, located in southern Iran. This region has consistently reported a high number of cases, making it one of the most affected areas in the country [8, 17, 18]. The prevalence of the disease in Fars Province is attributed to several factors, including the suitable climate and environmental conditions that favor the breeding and survival of sandflies and the vector responsible for transmitting the parasite [17, 19]. In addition, the presence of rural and agricultural communities in Fars Province increases the risk of exposure to infected sandflies.

Rodents serve as important reservoirs for CL in Iran [20–23]. Several species of rodents, including gerbils and jirds, have been identified as reservoirs of the *Leishmania* parasite. These rodents act as hosts for the parasite, allowing it to multiply and spread within their populations. The proximity of rodents to human settlements increases the risk of transmission, as infected sandflies can easily bite both humans and rodents [24, 25].

The existence of suitable environmental conditions for the life of rodents, especially gerbils, is the main reason for the endemicity of CL in southern Iran [8, 26–29]. Shiraz, located in the southern region of Iran, is a heavily populated city and serves as the capital of Fars Province. Until recent years, this city was considered one of the important foci of anthroponotic cutaneous leishmaniasis (ACL), but the epidemiological picture of the disease has changed in this city in recent years, in such a way that zoonotic cutaneous leishmaniasis (ZCL) has become the dominant form of the disease in this urban area [17, 18, 30–32].

Understanding the prevalence of CL in any endemic area is crucial. Equally important is having information related to the disease's reservoirs, agents, and vectors, as well as understanding the genetic diversity of the parasite. These elements are vital for implementing effective prevention and control measures.

The present study was designed and carried out to investigate the *L. major* infection of rodents, based on the molecular datasets of *Leishmania* kDNA and ITS2 genes, in an urban area (Shiraz city), in southern Iran.

## 2. Materials and Methods

### 2.1. Area of the Study and Sampling of Rodents

**Figure 1:**
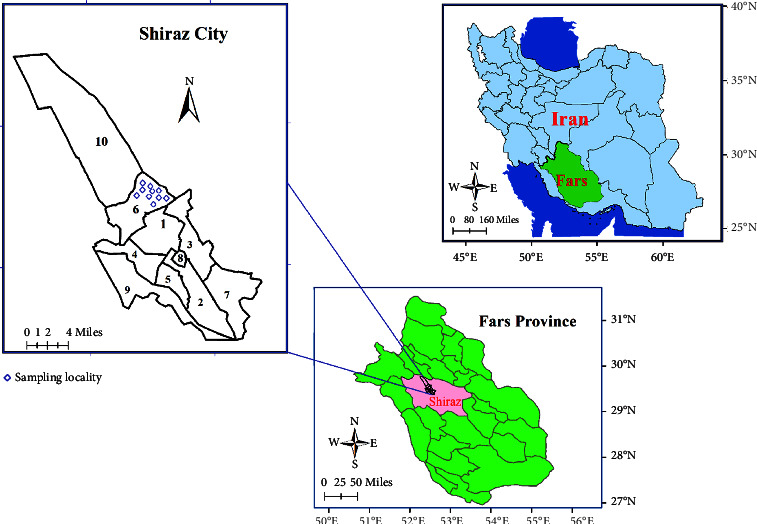
Map of Iran and Fars Province showing the sampling localities of rodents in Shiraz City (numbers are related to districts).

The study was carried out in Shiraz City, situated in Fars Province (Figure 1). Fars Province, located in the southern part of Iran, is one of the country's provinces. Shiraz, the provincial capital, is divided into 10 districts and is positioned between 29° and 30° North and 51.5°–52.5° East (Figure 1). The city is considered as the most populous city in this province and the fifth most populous city in the country [33].

All rodents were captured in 10 stations, in a newly emerged focus of cutaneous leishmaniasis in the northeast of Shiraz, District 6 (Figure 1). To capture rodents for the study, Sherman live traps were used and placed in gardens and houses within the city during the evening (Figure 1). To minimize the pain and distress of the rodents, they were euthanized with CO_2_ [34]. External measurements, sex, and other characteristics were recorded before dissection, and samples were taken from the spleen, liver, and skin. If there was a lesion in the skin, an impression smear sample was prepared from the skin lesion. All samples were kept at −20°C for subsequent DNA extraction.

### 2.2. Smear Preparation and DNA Extraction

For direct visualization of *Leishmania* amastigotes, multiple slides were prepared from the liver, spleen, sole, and any visible skin lesions of the captured rodents using the stamp-smear method. The smears were air-dried, fixed by methanol, and then stained with 10% Giemsa stain for 20 minutes and examined under a light microscope.

The Favorgen Biotech Corp. Kit (Taiwan) was used to extract total genomic DNA from tissue samples of rodents, including liver, spleen, and skin and patent lesions, according to the manufacturer's guidelines.

### 2.3. Polymerase Chain Reaction (PCR)

The LINR4 (5′-GGG GTT GGT GTA AAA TAG GG-3′) and LIN17 (5′-TTT GAA CGG GAT TTC TG-3′) primers [35] were used for the amplification of *Leishmania-*specific kDNA gene. By using these primers, 650-bp [35], 720-bp [35], and 760-bp [17, 28, 36–38] fragments of the kDNA of *L. major*, *L. infantum*, and *L. tropica* are, respectively, detectable. The PCR machine was programmed as follows: predenaturation (at 95°C for 5 min), denaturation (35 cycles, at 94°C for 30 s), annealing (at 52°C for 30 s), extension (at 72°C for 45 s), and a final extension (at 72°C for 8 min). A final volume of 25 *μ*L reaction, including 3.5 *μ*L of extracted DNA, 0.5 *μ*L (10 pm) of each primer, 12.5 *μ*L master mix (Ampliqon, Odense, Denmark), and 8 *μ*L of DW was used for PCR amplification [28].

For amplification of the *Leishmania* ITS2 gene, the primers of 5′-AAACTCCTC TCTGGTGCTTGC-3′ (forward) and 5′-AAACAAAGGTTGTCGGGGG-3′ (reverse) [39] were utilized. The length of the fragment amplified by these primers is 420 base pairs for *L. major*. The final volume (25 *μ*L) of PCR reactions included the following: extracted DNA (1 *μ*L, 100 ng/*μ*L), each primer (0.6 *μ*L, 10 pm), Taq DNA Polymerase Master Mix RED (12.5 *μ*L of 1x), and DW (10.3 *μ*L). The PCR machine was programmed as follows: initial denaturation (at 94.5°C for 5 min), denaturation (35 cycles at 94°C for 30 s), annealing (at 55°C for 30 s), extension (at 72°C for 30 s), followed by a final extension (at 72°C for 8 min) [28].

DNA electrophoresis was carried out on a 2% agarose gel for 45 min at 80 V by adding 3.5 *μ*L of the PCR products, a 100 bp molecular marker (SMOBIO, Hsinchu, Taiwan), and positive controls (reference strains of *L. infantum*, *L. tropica*, and *L. major*). The PCR products were sequenced for the ITS2 fragment, using the same pair of primers, used in the PCR assay.

### 2.4. Phylogenetic Analyses

The raw nucleotide sequences (forward and reverse directions) and chromatograms were checked and analyzed, and the consensus sequences were aligned, using Clustal W. The final sequences were registered in GenBank with accession numbers of ON398771, ON3987781-87, ON3987789, and ON3987892. The phylogenetic analysis involved 26 partial ITS2 gene sequences of *Leishmania* comprising 19 sequences of *L. major*. A total of 16 sequences were selected from the GenBank database (Table 1). The ITS2 sequence of *Crithidia mellificae* was considered as outgroup. Phylogenetic relationships between *Leishmania* species were reconstructed using a Bayesian inference (BI) tree in BEAST, version 2.6.7 (https://www.beast2.org/). The reliability of nodes was assessed using Bayesian posterior probability for the Bayesian. The neighbor-joining tree with 100000 bootstrap generations was conducted using the Kimura 2-parameter (K2P) model [46] in MEGA X software [47].

## 3. Results

### 3.1. Rodent Fauna and *Leishmania* Infection

**Figure 2:**
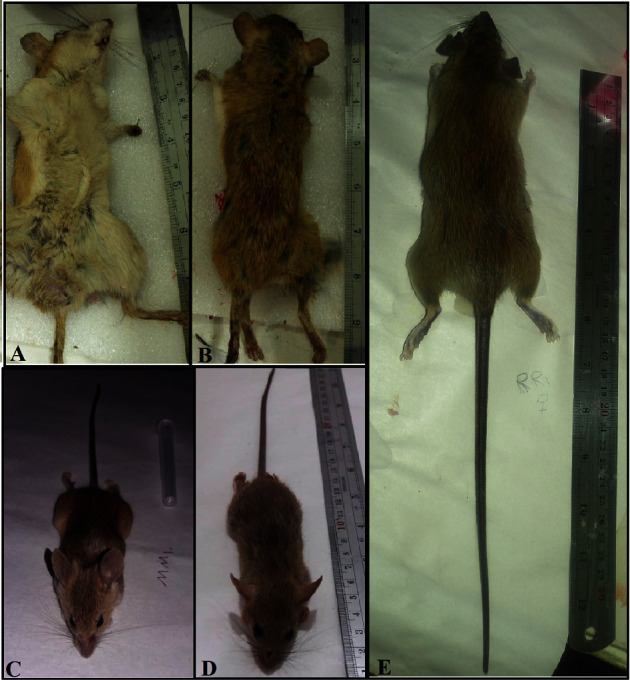
The captured rodents that were PCR-positive for *Leishmania* were *Meriones persicus* (A, B), *R. rattus* (D, E), and *Mus musculus* (C).

**Figure 3:**
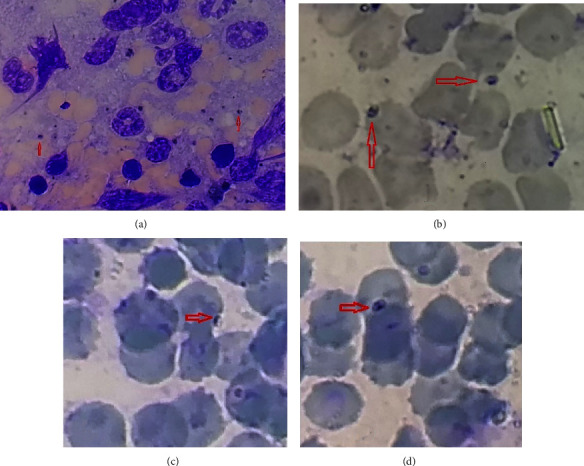
*Leishmania* amastigotes (arrows) are seen in the spleen smear of *M. musculus* (a, b) and *R. rattus* (c, d).

**Figure 4:**
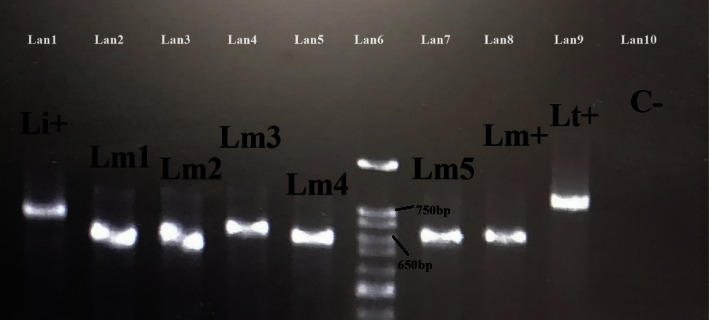
PCR-based amplification of kDNA gene. Bands on the electrophoresis gel are related to PCR of DNA extracted from the lesion (Lm1) or spleen tissue samples (Lm 2–5) of *R. rattus* (Lm1), *R. rattus* (Lm2), *M. musculus* (Lm3), and *Meriones persicus* (Lm5). Reference samples of *Leishmania infantum* (Li+), *L. tropica* (Lt+), and *L. major* (Lm+) and negative control (C−) were included.

A total of 41 rodents, belonging to three species were caught from 10 trapping stations. The trapping stations were in District 6 of Shiraz (Figure 1) which is not far from a densely populated area in the city. The caught rodent species were *Rattus rattu*s (*n* = 25, 60.97%), *Mus musculus* (*n* = 15, 36.58%), and *Meriones persicus* (*n* = 1, 2.5%). Amastigotes of *Leishmania* were seen in the spleen smear tissue of 6 (2.43%) of the (Figure 2) rodents, including 4 of *R. rattus* and 2 of *M. musculus* (Figure 3, Table 2). Skin lesions on the muzzle of two of *R. rattus* and one *Mus musculus* were seen. Samples that were taken from these lesions were PCR-positive for *Leishmania* (Figure 4).

Multiple lesions were seen simultaneously on the sole, lip, femur, and other parts of the body of one of the *R. rattus* rodents, while only the sample prepared from the sole was positive for *Leishmania* infection.

**Figure 5:**
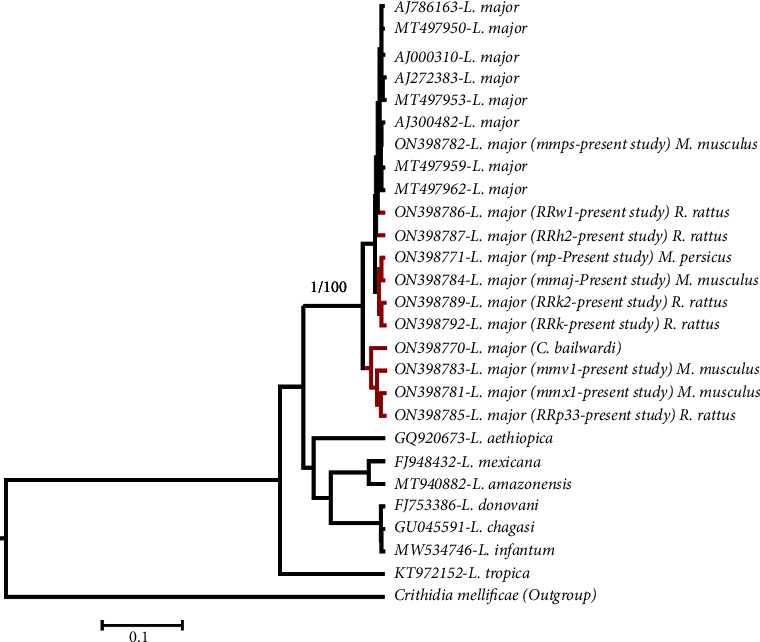
The evolutionary relationships of *Leishmania* species (ITS2 gene sequences) inferring based on the Bayesian method [48]. The posterior probability value for the Bayesian tree and the percentage value of bootstrap tests (10000 replicates) for the neighbor-joining tree are shown at the base of the clades before and after the slash symbol, respectively.

*Leishmania's* DNA was detected in 18 (43.9%) rodents, including 11 of *R. rattus*, 6 of *M. musculus*, and one of *M. persicus*, based on the PCR of kDNA (Figure 4) and sequencing of ITS2 gene (Figure 5). The *Leishmania* infection rate in *R. rattus* and *M. musculus* were 44% and 40%, respectively. Most (81.8%) of the *R. rattus* which were positive for *Leishmania* infection were male while most of the positive *M. musculus* (80%) cases were female (Table 2).

### 3.2. Phylogenetic Analysis

Phylogenetic analysis revealed the parasite infecting the rodents *R. rattus*, *M. persicus*, and *M. musculus* belongs to *L. major* (Figure 5). The *L. major* group was divided into two main clades with a bootstrap of 100 and posterior probability value of one (Figure 5). The average evolutionary divergence over sequence pairs was 0.0344 while within the *L. major* infecting rodents in the current study was 0.02.

## 4. Discussion

Leishmaniasis is one of the important parasitic diseases in different countries of the world, especially in tropical and subtropical regions. Iran is one of the important foci of leishmaniasis in the world, and every year a significant number of patients with cutaneous and visceral leishmaniasis are reported from this country [8, 17]. In the Fars Province in southern Iran, both CL and visceral leishmaniasis (VL) are present [49–55]. Vectors and animal reservoirs of CL are two main affective factors in the spread and emergence of the disease. Previous studies demonstrated ACL type of CL in the city of Shiraz, the capital of Fars Province, especially in the center of the city, while the ZCL is mostly reported from the rural areas in the province or from the outskirts of the city [13, 17, 30, 32]. Changes in the profile of CL have been previously reported in a study conducted by Davami et al. in one of the cities of the province, where ZCL has become the dominant form of the disease in the city [56, 57].

In this study, *Leishmania* infection was confirmed in rodents captured in urban gardens and residential areas, indicating a concerning shift in the epidemiology of leishmaniasis. This suggests that the disease, traditionally associated with rural areas, has now permeated into city centers.

The urbanization of ZCL is an escalating issue in numerous countries, including Iran [17, 27, 58, 59]. As urban areas continue to expand and encroach upon natural habitats, the risk of transmission and spread of the disease increases. This urban growth induces environmental changes that promote the proliferation of both sand fly vectors and reservoir rodents, thereby increasing the potential for disease spread.

One of the main factors contributing to the urbanization of CL is the construction of new settlements and infrastructure. These developments often disrupt natural ecosystems, leading to the displacement of wildlife and the introduction of new habitats for sandflies and their rodent hosts. In addition, the influx of people into urban areas can create overcrowded living conditions, poor sanitation practices, and limited access to healthcare, all of which contribute to the spread of the disease.

Another aspect of urbanization that plays a role in the transmission of CL is the increased movement of people and goods. Urban areas are often hubs for transportation and trade, facilitating the movement of infected individuals and potentially infected animals. This can lead to the introduction of new *Leishmania* strains into urban populations, further complicating control efforts.

The consequences of urbanization on CL are not limited to its transmission dynamics but also impact the burden of the disease on affected communities. Urban areas tend to have better healthcare infrastructure compared to rural areas, which may result in higher rates of diagnosis and reporting. However, the concentration of susceptible individuals in urban settings can also lead to larger outbreaks and more severe disease outcomes if control measures are not effectively implemented.

In Iran, *Rhombomys opimus* and *Meriones libycus* (Rodentia: Gerbillidae) serve as the main reservoir hosts for ZCL [20–22, 60]. The rodents of *Rhombomys opimus*, *T. indica*, *M. libycus*, and *Meriones hurrianae* have been reported as the major reservoir hosts of ZCL in a different area of Iran, while other species like *Nesokia indica* and *Gerbillus nanus* have been described as the accidental or probable reservoir hosts in different parts of Iran [20, 21, 61–64]. In Fars Province, the species of *M. libycus*, *M. persicus*, *T. indica*, *M. musculus*, and *R. rattus* have been found to be positive for *Leishmania* infection [20–22, 26, 27, 60–62, 65, 66]. In a recent study, we also reported the infection of Calomyscid rodents with *L. major* in the mountainous area of the same area of the current study [28]. In recent years *Leishmania* infection in *Mus musculus*, commonly known as the house mouse, has been reported from different areas of the world including Iran [23, 64, 67, 68]. One study by Parhizkari and colleagues investigated the role of rodents caught in southern Iran as the reservoir hosts for *L. major*. The researchers collected *Mus musculus* from different habitats and examined them for the presence of *Leishmania* parasites. They found that a high proportion of mice (42.9%) were infected with *L. major*, indicating their importance as a possible reservoir host [23]. Moreover, vertical transmission of *L. infantum* in *Mus musculus* has been recently documented [69].

In the present study, *R. rattus* was the most abundant rodent species infected with *L. major*. Although rodents in the subfamily Gerbillinae are the most likely reservoir hosts in the rural area of Fars Province, it seems that rodents of the genera *rattus* and *Mus* are important hosts or probably reservoirs of *L. major* in the transmission of CL in the urban area, in Fars Province.

The newly identified focus of ZCL, as described in this study, is situated in a relatively mountainous and foothill region on the periphery of a densely populated area in the city of Shiraz. This area provides an ideal habitat for the Persian jird (*M. persicus*), which is recognized as a probable reservoir of ZCL in Iran [70]. Therefore, it is not unexpected that *L. major* infection was also detected in this species during our study.

In our research, we not only detected *Leishmania* molecularly but also observed *Leishmania* amastigotes in tissue slides prepared from the spleens of infected *R. rattus* and *M. musculus*. This could serve as evidence for the potential transmission of *L. major* from these rodents to the parasite vector *Ph. papatasi* and subsequently to humans. A comprehensive investigation is required to ascertain the role of these rodents in transmitting *Leishmania* to sandflies. This could help clarify the uncertainties surrounding the role of these synanthropic rodents in the epidemiology of ZCL in this region of Iran.

## 5. Conclusion

The findings of the current study reveal that rodents captured in urban areas of Fars Province, southern Iran, are infected with *L. major*. Notably, the infection of *Mus musculus* with this parasite has been confirmed in this study. The presence of *Leishmania* infection in these rodents in urban areas signals the potential urbanization of ZCL, caused by *L. major*. This urbanization presents unique challenges for control and prevention efforts. The increased proximity of humans, rodents, and sandflies in urban areas amplifies the risk of transmission. To effectively combat this disease, a multidisciplinary approach is required, focusing on surveillance, vector control, public awareness, and collaboration between different sectors. Only through these concerted efforts can the urbanization of CL be effectively addressed and its impact minimized.

## Figures and Tables

**Table 1:** Information about the ITS2 sequences of *Leishmania* used for the phylogenetic analyses.

Species	Host	Country	Accession number	Reference
*L. major*	*Mus musculus*	Iran	ON3987781-84	Present study
	*Rattus rattus*	Iran	ON3987785-87, ON3987789, ON3987892	Present study
	*Meriones persicus*	Iran	ON398771	Present study
	*Calomyscus cf. bailwardi*	Iran	ON398770	[28]
	Human	Kenya	AJ300482	—
	Human	Turkmenistan	AJ272383	—
	Human	Russia	AJ000310	—
	Human	Spain	MT497962	[40]
	Human	Spain	MT497950	[40]
	Human	Spain	MT497959	[40]
	Human	Spain	MT497953	[40]
	Human	Iran	AJ786163	[41]

*L. aethiopica*	Human	USA (CDC)	GQ920673	[42]

*L. mexicana*	Human	USA (CDC)	FJ948432	[42]

*L. chagasi*	Human	USA (CDC)	GU045591	[42]

*L. infantum*	Sand fly	Sudan	MW534746	[43]

*L. donovani*	Human	USA (CDC)	FJ753386	[42]

*L. tropica*	Human	Azerbaijan	KT972152	[44]

*L. amazonensis*	Human	Brazil	MT940882	[45]

Centers for disease control and prevention (CDC).

**Table 2:** Molecular and microscopic characterization of *L. major* infection in captured rodents. The parameters considered include species, sex, PCR (kDNA) test results, presence of lesions, and observation of *L. major* amastigotes in the rodent tissues.

Species	Sex	L.m. amastigote	PCR			Lesion observation
			Liver	Spleen	Sole	
*R. rattus*	Male	−	−	+	−	N
*R. rattus*	Male	Spleen	+	+	−	N
*R. rattus*	Female	−	−	+	−	N
*R. rattus*	Male	Spleen	−	+	−	N
*R. rattus*	Male	Spleen	−	+		Muzzle
*R. rattus*	Male	−	+	+	−	N
*R. rattus*	Male	−		+	−	N
*R. rattus*	Male	Spleen	−	+	−	Body/foot/lip/muzzle
*R. rattus*	Male	−	−	+	−	N
*R. rattus*	Male	−	−	+	+	N
*R. rattus*	Female	−	−	+	−	N
*M. musculus*	Male	−	−	+	−	N
*M. musculus*	Female	Spleen	+	+	−	N
*M. musculus*	Female	−	−	+	−	N
*M. musculus*	Female	−	−	+	−	N
*M. musculus*	Female	Spleen	−	+		Muzzle
*M. musculus*	Male	−	+	+	−	N
*Meriones persicus*	Female	−	−	+	−	N

Positive infection (+), negative infection (−), not seen (N), L. major (L.m.).

## Data Availability

The data used to support the findings of this study are included in the article.

## References

[1] Sangenito L. S., da Silva Santos V., d’Avila-Levy C. M., Branquinha M. H., Souza dos Santos A. L., de Oliveira S. S. (2019). Leishmaniasis and Chagas disease–neglected tropical diseases: treatment updates. *Current Topics in Medicinal Chemistry*.

[2] Read A., Hurwitz I., Durvasula R. (2012). Leishmaniasis: an update on a neglected tropical disease. *Dynamic Models of Infectious Diseases: Volume 1: Vector-Borne Diseases*.

[3] Who (2022). https://www.who.int/news-room/fact-sheets/detail/leishmaniasis.

[4] Loría-Cervera E. N., Andrade-Narváez F. J. (2014). Animal models for the study of leishmaniasis immunology. *Revista do Instituto de Medicina Tropical de São Paulo*.

[5] Kumar R., Engwerda C. (2014). Vaccines to prevent leishmaniasis. *Clinical and translational immunology*.

[6] Hotez P. J., Savioli L., Fenwick A. (2012). Neglected tropical diseases of the Middle East and North Africa: review of their prevalence, distribution, and opportunities for control. *Public Library of Science Neglected Tropical Diseases*.

[7] Knight C. A., Harris D. R., Alshammari S. O., Gugssa A., Young T., Lee C. M. (2022). Leishmaniasis: recent epidemiological studies in the Middle East. *Frontiers in Microbiology*.

[8] Sabzevari S., Teshnizi S. H., Shokri A., Bahrami F., Kouhestani F. (2021). Cutaneous leishmaniasis in Iran: a systematic review and meta-analysis. *Microbial Pathogenesis*.

[9] Ul Bari A. (2006). Epidemiology of cutaneous leishmaniasis. *Journal of Pakistan Association of Dermatologists*.

[10] Firooz A., Mortazavi H., Khamesipour A. (2021). Old world cutaneous leishmaniasis in Iran: clinical variants and treatments. *Journal of Dermatological Treatment*.

[11] Hezari F., Niyyati M., Seyyed Tabaei S. J. (2016). Frequency of cutaneous leishmaniasis and species identification in suspected individuals from golestan province, northern Iran in 2014. *Iranian Journal of Public Health*.

[12] Teimouri A., Mohebali M., Kazemirad E., Hajjaran H. (2018). Molecular identification of agents of human cutaneous leishmaniasis and canine visceral leishmaniasis in different areas of Iran using internal transcribed spacer 1 PCR-RFLP. *Journal of Arthropod-Borne Diseases*.

[13] Sarkari B., Ahmadpour N. B., Motazedian M. H. (2016). Inter- and intraspecific variations of Leishmania strains isolated from patients with cutaneous and visceral leishmaniases in fars province, south of Iran. *Iranian Journal of Medical Sciences*.

[14] Rasti S., Ghorbanzadeh B., Kheirandish F. (2016). Comparison of molecular, microscopic, and culture methods for diagnosis of cutaneous leishmaniasis. *Journal of Clinical Laboratory Analysis*.

[15] Okwor I., Uzonna J. (2016). Social and economic burden of human leishmaniasis. *The American Journal of Tropical Medicine and Hygiene*.

[16] Galvão E. L., Pedras M. J., Cota G. F., Rabello A., Simões T. C. (2019). How cutaneous leishmaniasis and treatment impacts in the patients’ lives: a cross-sectional study. *Public Library of Science One*.

[17] Davoodi T., Khamesipour A., Shahabi S., Gholizadeh F., Pourkamal D., Hatam G. (2022). Geographical distribution and molecular epidemiology of cutaneous leishmaniasis in Fars Province, southern Iran. *Environmental Science and Pollution Research*.

[18] Khosravani M., Nasiri Z., Keshavarz D., Rafat-Panah A. (2016). Epidemiological trend of cutaneous leishmaniasis in two endemic focus of disease, south of Iran. *Journal of Parasitic Diseases*.

[19] Yaghoobi-Ershadi M. (2012). Phlebotomine sand flies (Diptera: psychodidae) in Iran and their role on Leishmania transmission. *Journal of arthropod-borne diseases*.

[20] Azizi K., Davari B., Kalantari M., Fekri S. (2011). Gerbillid rodents fauna (Muridae: Gerbillinae) and detection of reservoir hosts (s) of zoonotic cutaneous leishmaniasis using a nested-PCR technique in Jask City in Hormozgan Province in 2008. *Scientific Journal of Kurdistan University of Medical Sciences*.

[21] Azizi K., Moemenbellah-Fard M. D., Kalantari M., Fakoorziba M. R. (2012). Molecular detection of Leishmania major kDNA from wild rodents in a new focus of zoonotic cutaneous leishmaniasis in an oriental region of Iran. *Vector Borne and Zoonotic Diseases*.

[22] Mohebali M., Javadian E., Yaghoobi Ershadi M., Akhavan A., Hajjaran H., Abaei M. (2004). Characterization of Leishmania infection in rodents from endemic areas of the Islamic Republic of Iran. *Eastern Mediterranean Health Journal*.

[23] Parhizkari M., Motazedian M., Asqari Q., Mehrabani D., Parasitology (2011). The PCR-based detection of Leishmania major in *Mus musculus* and other rodents caught in southern Iran: a guide to sample selection. *Annals of Tropical Medicine and Parasitology*.

[24] Lainson R. (1988). Ecological interactions in the transmission of the leishmaniases. *Philosophical Transactions of the Royal Society of London- Series B: Biological Sciences*.

[25] Gratz N. G. (2018). Rodents and human disease: a global appreciation. *Rodent Pest Management*.

[26] Moemenbellah-Fard M., Kalantari M., Rassi Y., Javadian E. (2003). The PCR-based detection of Leishmania major infections in Meriones libycus (Rodentia: muridae) from southern Iran. *Annals of Tropical Medicine and Parasitology*.

[27] Motazedian M. H., Parhizkari M., Mehrabani D., Hatam G., Asgari Q. (2010). First detection of Leishmania major in *Rattus norvegicus* from Fars province, southern Iran. *Vector Borne and Zoonotic Diseases*.

[28] Shahabi S., Azizi K., Asgari Q., Sarkari B. (2023). Calomyscid rodents (rodentia: calomyscidae) as a potential reservoir of zoonotic cutaneous leishmaniasis in a mountainous residential area in the plateau of Iran: inferring from molecular data of kDNA and ITS2 genes of Leishmania major. *Journal of Tropical Medicine*.

[29] Bamorovat M., Sharifi I., Aflatoonian M. R. (2024). A prospective longitudinal study on the elimination trend of rural cutaneous leishmaniasis in southeastern Iran: climate change, population displacement, and agricultural transition from 1991 to 2021. *Science of the Total Environment*.

[30] Barazesh A., Motazedian M. H., Fouladvand M. (2019). Molecular identification of species caused cutaneous leishmaniasis in southern zone of Iran. *Journal of Arthropod-Borne Diseases*.

[31] Khosravani M., Moemenbellah-Fard M. D., Sharafi M., Rafat-Panah A. (2016). Epidemiologic profile of oriental sore caused by Leishmania parasites in a new endemic focus of cutaneous leishmaniasis, southern Iran. *Journal of Parasitic Diseases*.

[32] Oryan A., Shirian S., Tabandeh M. R., Hatam G. R., Randau G., Daneshbod Y. (2013). Genetic diversity of Leishmania major strains isolated from different clinical forms of cutaneous leishmaniasis in southern Iran based on minicircle kDNA. *Infection, Genetics and Evolution*.

[33] Sabet Sarvestani M., Ibrahim A. L., Kanaroglou P. (2011). Three decades of urban growth in the city of Shiraz, Iran: a remote sensing and geographic information systems application. *Cities*.

[34] Boivin G. P., Hickman D. L., Creamer-Hente M. A., Pritchett-Corning K. R., Bratcher N. A. (2017). Review of CO₂ as a euthanasia agent for laboratory rats and mice. *Journal of the American Association for Laboratory Animal Science: Journal of the American Association for Laboratory Animal Science*.

[35] Aransay A. M., Scoulica E., Tselentis Y. (2000). Detection and identification of Leishmania DNA within naturally infected sand flies by seminested PCR on minicircle kinetoplastic DNA. *Applied and Environmental Microbiology*.

[36] Azizi K., Rassi Y., Javadian E., Motazedian M., Asgari Q., Yaghoobi-Ershadi M. (2008). First detection of Leishmania infantum in phlebotomus (larroussius) major (Diptera: psychodidae) from Iran. *Journal of Medical Entomology*.

[37] Mohammadpour I., Motazedian M. H., Handjani F., Hatam G. R. (2017). Lip leishmaniasis: a case series with molecular identification and literature review. *Bone Marrow Concentrate Infectious Diseases*.

[38] Azizi K., Kalantari M., Motazedian M., Asgari Q., Soltani A., Mohammadpour I. (2020). DNA-based detection of Leishmania and Crithidia species isolated from humans in cutaneous and post-kala-azar dermal leishmaniasis from Shiraz and Kharameh, southern Iran. *Journal of Vector Borne Diseases*.

[39] Mohebali M., Malmasi A., Khodabakhsh M. (2017). Feline leishmaniosis due to Leishmania infantum in Northwest Iran: the role of cats in endemic areas of visceral leishmaniosis. *Veterinary Parasitology: Regional Studies and Reports*.

[40] Fernández‐Arévalo A., Ballart C., Muñoz‐Basagoiti J. (2022). Autochthonous and imported tegumentary leishmaniasis in Catalonia (Spain): aetiological evolution in the last four decades and usefulness of different typing approaches based on biochemical, molecular and proteomic markers. *Transboundary and Emerging Diseases*.

[41] Mahnaz T., Katrin K., Amer A.-J. (2006). Leishmania major: genetic heterogeneity of Iranian isolates by single-strand conformation polymorphism and sequence analysis of ribosomal DNA internal transcribed spacer. *Acta Tropica*.

[42] de Almeida M. E., Steurer F. J., Koru O., Herwaldt B. L., Pieniazek N. J., da Silva A. J. (2011). Identification of Leishmania spp. by molecular amplification and DNA sequencing analysis of a fragment of rRNA internal transcribed spacer 2. *Journal of Clinical Microbiology*.

[43] Jaffe C. L., Baneth G., Abdeen Z. A., Schlein Y., Warburg A. (2004). Leishmaniasis in Israel and the Palestinian Authority. *Trends in Parasitology*.

[44] Khan N. H., Messenger L. A., Wahid S., Sutherland C. J. (2016). Phylogenetic position of Leishmania isolates from khyber pakhtunkhwa province of Pakistan. *Experimental Parasitology*.

[45] Coser E. M., Ferreira B. A., Yamashiro-Kanashiro E. H., Lindoso J. A. L., Coelho A. C. (2021). Susceptibility to paromomycin in clinical isolates and reference strains of Leishmania species responsible for tegumentary leishmaniasis in Brazil. *Acta Tropica*.

[46] Kimura M. (1980). A simple method for estimating evolutionary rates of base substitutions through comparative studies of nucleotide sequences. *Journal of Molecular Evolution*.

[47] Kumar S., Stecher G., Li M., Knyaz C., Tamura K. (2018). Mega X: molecular evolutionary genetics analysis across computing platforms. *Molecular Biology and Evolution*.

[48] Sneath P. H., Sokal R. R. (1973). *Numerical Taxonomy. The Principles and Practice of Numerical Classification*.

[49] Rezaei Z., Azarang E., Shahabi S., Omidian M., Pourabbas B., Sarkari B. (2020). Leishmania ITS1 is genetically divergent in asymptomatic and symptomatic visceral leishmaniasis: results of a study in southern Iran. *Journal of Tropical Medicine*.

[50] Layegh Gigloo A., Sarkari B., Rezaei Z., Hatam G. R., Davami M. H. (2018). Asymptomatic Leishmania infected children: a seroprevalence and molecular survey in a rural area of Fars Province, Southern Iran. *Journal of Tropical Medicine*.

[51] Mohebali M. (2013). Visceral leishmaniasis in Iran: review of the epidemiological and clinical features. *Iranian Journal of Parasitology*.

[52] Rezaei Z., Sarkari B., Dehghani M., Layegh Gigloo A., Afrashteh M. (2018). High frequency of subclinical Leishmania infection among HIV-infected patients living in the endemic areas of visceral leishmaniasis in Fars province, southern Iran. *Parasitology Research*.

[53] Sarkari B., Gadami F., Shafiei R. (2015). Seroprevalence of Leishmania infection among the healthy blood donors in kala-azar endemic areas of Iran. *Journal of Parasitic Diseases*.

[54] Sarkari B., Hatam G., Ghatee M. (2012). Epidemiological features of visceral leishmaniasis in fars province, southern Iran. *Iranian Journal of Public Health*.

[55] Sarkari B., Naraki T., Ghatee M. A., Abdolahi Khabisi S., Davami M. H. (2016). Visceral leishmaniasis in southwestern Iran: a retrospective clinico-hematological analysis of 380 consecutive hospitalized cases (1999-2014). *Public Library of Science One*.

[56] Davami M. H., Motazedian M. H., Sarkari B. (2010). The changing profile of cutaneous leishmaniasis in a focus of the disease in Jahrom district, southern Iran. *Annals of Tropical Medicine and Parasitology*.

[57] Hosseini G., Sarkari B., Moshfe A., Motazedian M. H., Abdolahi Khabisi S. (2015). Epidemiology of human fascioliasis and intestinal helminthes in rural areas of boyer-ahmad township, southwest Iran; A population based study. *Iranian Journal of Public Health*.

[58] Caldart E. T., Freire R. L., Ferreira F. P. (2017). Leishmania in synanthropic rodents (*Rattus rattus*): new evidence for the urbanization of Leishmania (Leishmania) amazonensis. *Revista Brasileira de Parasitologia Veterinaria*.

[59] Organization W. H. (2002). Urbanization: an increasing risk factor for leishmaniasis. *Weekly Epidemiological Record= Relevé épidémiologique hebdomadaire*.

[60] Masoumeh A., Kourosh A., Mohsen K. (2014). Laboratory based diagnosis of leishmaniasis in rodents as the reservoir hosts in southern Iran. *Asian Pacific Journal of Tropical Biomedicine*.

[61] Kalantari M., Azizi K., Askari M., Sarkari B., Turki H. (2017). Acomys dimidiatus (Rodentia: muridae): probable reservoir host of Leishmania major, southern Iran. *Annals of Tropical Medicine and Public Health*.

[62] Mehrabani D., Motazedian M. H., Asgari Q., Hatam G. R., Owji S. A. A., Oryan A. (2011). Leishmania Major in Tatera Indica in Estahban, Southern Iran: Microscopy, Culture, Isoenzyme and PCR. *Pakistan Journal of Medical Sciences*.

[63] Pourmohammadi B., Motazedian M., Kalantari M. (2008). Rodent infection with Leishmania in a new focus of human cutaneous leishmaniasis, in northern Iran. *Annals of Tropical Medicine and Parasitology*.

[64] Yaghoobi-Ershadi M., Akhavan A., Mohebali M. (1996). Meriones Iibycus and Rhombomys opimus (Rodentia: Gerbillidae) are the main reservoir hosts in a new focus of zoonotic cutaneous leishmaniasis in Iran. *Transactions of the Royal Society of Tropical Medicine and Hygiene*.

[65] Azizi K., Moemenbellah-Fard M., Fakoorziba M., Fekri S. (2011). Gerbillus nanus (Rodentia: muridae): a new reservoir host of Leishmania major. *Annals of Tropical Medicine and Parasitology*.

[66] Parhizkari M., Motazedian M., Asqari Q., Mehrabani D. (2011). The PCR-based detection of Leishmania major in *Mus musculus* and other rodents caught in southern Iran: a guide to sample selection. *Annals of Tropical Medicine and Parasitology*.

[67] de Freitas T. P., D’Andrea P. S., de Paula D. A. (2012). Natural infection of Leishmania (viannia) braziliensis in *Mus musculus* captured in mato grosso, Brazil. *Vector Borne and Zoonotic Diseases*.

[68] Helhazar M., Leitão J., Duarte A., Tavares L., da Fonseca I. P. (2013). Natural infection of synathropic rodent species *Mus musculus* and *Rattus norvegicus* by Leishmania infantum in Sesimbra and Sintra--Portugal. *Parasites and Vectors*.

[69] Martín-Sánchez J., Torres-Medina N., Corpas-López V., Morillas-Márquez F., Díaz-Sáez V. (2020). Vertical transmission may play a greater role in the spread of Leishmania infantum in synanthropic *Mus musculus* rodents than previously believed. *Transboundary and Emerging Diseases*.

[70] Edrissian G. H., Ghorbani M., Tahvildar-Bidruni G. (1975). Meriones persicus, another probable reservoir of zoonotic cutaneous leishmaniasis in Iran. *Transactions of the Royal Society of Tropical Medicine and Hygiene*.

